# Novel Strategies for the Prevention and Treatment of Biofilm Related Infections

**DOI:** 10.3390/ijms140918488

**Published:** 2013-09-06

**Authors:** Meng Chen, Qingsong Yu, Hongmin Sun

**Affiliations:** 1 Nanova, Inc. Columbia, MO 65211, USA; E-Mail: chenmeng@nanovamed.com or mengchen2002slc@yahoo.com; 2 Department of Mechanical and Aerospace Engineering, University of Missouri, Columbia, MO 65211, USA; E-Mail: yuq@missouri.edu; 3 Division of Cardiovascular Medicine, Department of Internal Medicine, University of Missouri, Columbia, MO 65212, USA

**Keywords:** biofilm, antimicrobial, small molecule, biomaterial

## Abstract

Biofilm formation by human bacterial pathogens on implanted medical devices causes major morbidity and mortality among patients, and leads to billions of dollars in healthcare cost. Biofilm is a complex bacterial community that is highly resistant to antibiotics and human immunity. As a result, novel therapeutic solutions other than the conventional antibiotic therapies are in urgent need. In this review, we will discuss the recent research in discovery of alternative approaches to prevent or treat biofilms. Current anti-biofilm technologies could be divided into two groups. The first group focuses on targeting the biofilm forming process of bacteria based on our understanding of the molecular mechanism of biofilm formation. Small molecules and enzymes have been developed to inhibit or disrupt biofilm formation. Another group of anti-biofilm technologies focuses on modifying the biomaterials used in medical devices to make them resistant to biofilm formation. While these novel anti-biofilm approaches are still in nascent phases of development, efforts devoted to these technologies could eventually lead to anti-biofilm therapies that are superior to the current antibiotic treatment.

## 1. Introduction

Biofilm is a community of bacteria that are attached to a substratum or surface. Bacteria in biofilm are embedded in extracellular polymeric matrix produced by the bacteria. Bacteria develop biofilm on submerged surfaces such as natural aquatic systems, water pipes, living tissues, tooth surfaces, indwelling medical devices and implants [1]. Biofilm formation on indwelling medical devices and implants such catheters, mechanical heart valves, pacemakers, prosthetic joints, and contact lenses pose a critical medical problem. Both Gram-negative and Gram-positive bacteria can form biofilms on indwelling medical devices. The most common biofilm-forming bacteria include *Enterococcus faecalis*, *Staphylococcus aureus*, *Staphylococcus epidermidis*, *Streptococcus viridans*, *Escherichia coli*, *Klebsiella pneumoniae*, *Proteus mirabilis*, and *Pseudomonas aeruginosa* [2].

Among these biofilm-forming bacteria, *S. aureus* and *S. epidermidis* are most commonly found on cardiovascular devices [3,4]. It was estimated that *S. aureus* and *S. epidermidis* caused about 40%–50% of prosthetic heart valve infections, and 50%–70% catheter biofilm infections [5]. Each year about 250,000–500,000 primary blood stream infections occur among the 150 million intravascular devices implanted in the US. Health care cost could be increased from $4000 to $56,000 for each infection [6,7]. Approximately 87% of blood stream infections were caused by staphylococci [5]. Taken together, the burden on healthcare system by *S. aureus* and *S. epidermidis* in biofilm is enormous.

Biofilm formation is initiated when bacterial cells attach and adhere to the surfaces of implants or host tissues. *S. aureus* generates multiple adhesive factors that could bind to host factors [8]. The host factors could mediate bacterial attachment to implant surfaces, which is covered by host plasma and other extracellular components. For example, *S. aureus* produces fibronectin-binding proteins (FnBPA and FnBPB) [9], collagen-binding protein Cna [10] and fibrinogen-binding proteins, clumping factor A and B (ClfA and ClfB) [11,12] to bind host plasma and extracellular matrix (ECM) components. Bacteria attached to the surfaces will proliferate, aggregate and recruit cells from the surrounding to form and differentiate into biofilm structures [13]. Bacterial attachment to the surface will change from reversible to irreversible accompanied by profound physiological, gene expression and protein profile changes. The mature biofilm structures consist of complex architecture and channels. Bacterial cells can detach from mature biofilms and spread to other organ systems [13,14]. As a result, biofilms become sources of persistent and chronic infections.

Bacteria in biofilm behave differently from planktonic bacteria, especially in terms of their response to antibiotic treatment [2]. Biofilm-associated bacteria are highly resistant to antibiotics. The complicated structure of biofilm with extracellular polymeric matrix could prevent antibiotics from reaching the bacteria. Bacteria in biofilm could also adopt a slow growing or starved state due to the altered microenvironment such as depletion of nutrition and accumulation of waste. The changed physiological state of bacteria could make them more resistant to antibiotics, which target more active cell processes [3,15–17].

In addition to the difficulty of treating biofilm with conventional antibiotic therapy, treating biofilm is further hindered by the rising antibiotic resistance among pathogens. Antibiotics targets are essential for bacterial survival. As a result, antibiotic resistant strains have been favored by selective pressure [18]. Antibiotic resistance in *S. aureus* such as the methicillin resistance is one of the most urgent medical problems [19,20]. It was estimated that 94,360 invasive methicillin-resistant *Staphylococcus aureus* (MRSA) infections occurred in the US in 2005, and these infections were associated with death in 18,650 cases [21]. Although *S. epidermidis* is part of the normal human epithelial bacterial flora, it can cause infection when skin or mucous membrane is injured. Biofilm formation on implanted indwelling medical devices is the major manifestation of *S. epidermidis* pathogenesis [3]. Antibiotic resistance is also widespread in *S. epidermidis.* For example, more than 70% of all hospital isolates of *S. epidermidis* are resistant to methicillin [22]. In summary, alternative approaches other than conventional antibiotic therapy are in urgent need to treat biofilm related infections. In this review, we will discuss alternative approaches to prevent or treat biofilms focusing on *S. aureus* and *S. epidermidis*, two of the most important biofilm forming pathogens.

## 2. Anti-Biofilm Agents

### 2.1. Small Molecules

High throughput screening of small molecule libraries has been one of the major approaches to search for drug leads. In recent years, high throughput screening has been increasingly adopted in academics to screen for low molecular weight compounds with desired biological properties. A chemical series of small compounds was identified by our group that inhibited the virulence gene expression of Gram positive pathogens such as *Streptococcus pyogenes* and *S. aureus* [23,24].

We performed a high throughput screening of 55,000 chemical compounds to search for inhibitors of gene expression of a key *S. pyogenes* virulence factor streptokinase [24]. A lead compound and its analogs were identified to be able to inhibit streptokinase gene expression. Detailed analysis of the global effect of the inhibitor on *S. pyogenes* gene expression demonstrated that the inhibitor changed gene expression of many key virulence factors. Furthermore, the lead compound also protected mice against *S. pyogenes* infection [24]. Analogs of the lead compounds were subsequently tested in *S. aureus.* Two analogs from the same chemical series inhibited biofilm formation by *S. aureus* [23]. The anti-biofilm compound also inhibited gene expression of a number of important *S. aureus* virulence factors [23]. Among the inhibited genes are genes known to be involved in biofilm formation. Inhibition of these genes could lead to inhibition of biofilm formation. The broad spectrum anti-virulence effect of the compounds on both *S. pyogenes* and *S. aureus* suggested that this class of compounds could target a conserved gene regulatory mechanism. As a result, this class of compounds could potentially be developed into novel anti-microbial agents against multiple pathogens.

Panmanee *et al.* screened 42,865 compounds to identify compounds that inhibited formation of or kill *S. epidermidis* biofilms. Sixteen compounds were confirmed to be able to either kill or inhibit *S. epidermidis* biofilm [25]. The mechanism of action of these anti-biofilm compounds remained to be characterized. Sambanthamoorthy *et al.* performed high throughput screening on 66,000 compounds and natural products to identify small molecules that inhibited induction of *Vibrio cholerae* cyclic di-GMP-inducible transcription [26]. Cyclic di-GMP is a second-messenger signal that is a key regulator of switch between planktonic and attached lifestyle of the majority of bacteria [27,28]. A benzimidazole compound demonstrated broad spectrum inhibition of biofilm formation by several Gram-negative and Gram-positive bacterial pathogens, including *P. aeruginosa* and *S. aureus* [26]. Opperman *et al.* screened 87,250 compounds for inhibitors of *S. epidermidis* biofilm [29]. Twenty three aryl rhodanines were identified to inhibit early phase biofilm formation by multiple strains of *S. aureus*, *S. epidermidis*, and *E. faecalis* [29]. However, the mechanism underlying the function of the aryl rhodanines was unclear.

*P. aeruginosa* produces an organic compound cis-2-decenoic acid capable of dispersing established biofilms and inhibiting biofilm development by a number of bacteria [30]. It was observed that *P. aeruginosa* dispersed from a continuous culture biofilm after medium flow stopped for several hours. The extracellular message that induced the release of cells from biofilm was purified from the organic fraction of spent medium and identified as *cis*-2-decenoic acid which was able to disperse biofilms by *E. coli*, *K. pneumoniae*, *P. mirabilis*, *S. pyogenes*, *B. subtilis*, *S. aureus*, and *C. albicans* [30]. Similarly, bacteria produce d-amino acids, which inhibited biofilm formation by *S. aureus* and *P. aeruginosa* [31].

*N*-acetylcysteine is a mucolytic agent that could interfere with exopolysaccharide formation in biofilms and inhibit *S. epidermidis* biofilm formation [32]. Metallic cations such as Ca^2+^ and Mg^2+^ play roles in microbial adherence and biofilm formation. As a result, chelators that can remove these cations could also inhibit biofilm formation [33].

The number of small molecules that can interfere with biofilm formation and thus serve as lead for development of anti-biofilm agents is growing rapidly (Table 1). However, mechanisms of action of many of these small molecules are still unclear which hinders the further development. More pharmacokinetic and *in vivo* studies are needed to optimize these leads to meet the necessary criteria for medical application.

### 2.2. Matrix-Targeting Enzymes

Disrupting or degrading the extracellular polymeric matrix of biofilms can weaken and disperse biofilms. There have been a number of studies done to degrade matrix components such as polysaccharide, eDNA and proteins [34]. The Gram-negative, oral bacterium *Actinobacillus actinomycetemcomitans* produces dispersin B that could disperse biofilms by other bacteria. Kaplan *et al.* found that dispersin B could disrupt extracellular matrix of *S. epidermidis* biofilm and disperse the biofilm [35]. Extracellular genomic DNA (eDNA) is released by bacteria as an important component of extracellular matrix of biofilm [36]. As a result, DNase I was shown to be able to disperse *S. aureus* biofilms [37]. Proteinase K and trypsin effectively disrupted *S. aureus* biofilms [38]. There are still a lot of limitations with these approaches. The *in vivo* efficacy of such approaches isn’t well established and treating host with proteins could cause inflammatory and allergic reaction, which could affect the therapeutic potential [38].

## 3. Bioengineering Approaches

### 3.1. Bactericidal/Bacteriostatic Coating

Altering the surface properties of indwelling medical devices is one of the main focuses to prevent or decrease biofilm infections [3,39]. One of the approaches to make biomaterial surfaces resistant to biofilm formation is to coat the surface with bactericidal/bacteriostatic substances. Antibiotics are commonly used. For example, vancomycin was covalently bonded to the surface of titanium metal implant. As a result, *S. epidermidis* biofilm formation was significantly inhibited on a vancomycin coated titanium alloy [40]. Antibiotics have been used to impregnate catheters to prevent biofilm formation in clinics [41–44]. However, using antibiotics could lead to selection of antibiotic resistance and even induce biofilm formation [45].

Heavy metal silver was also used as an anti-biofilm agent by depositing silver on the surfaces of biomaterials using coating technology [46,47]. Silver is one of the strongest bactericidal agents. The mechanism of the bactericidal function of silver is still unclear. It was observed that when silver ion penetrated into cells, DNA was condensed and lost ability to replicate, which led to cell death. Silver ion could also inactivate proteins by reacting with the thiol groups in cysteine residues [48–50]. Silver nanoparticles have been studied for their antimicrobial property. Because silver nanoparticles have extremely large surface area, they can interact with microorganisms better. The nanoparticles could penetrate inside the bacteria and react with proteins and DNA, and interrupt the respiratory chain and cell division, leading to cell death [48].

Coating medical devices with silver ions or metallic silver has disappointing clinic results, probably due to inactivation of metallic silver when the devices contacting blood and coating wearing off [48]. On the other hand, biofilm formation by a number of pathogens such *E. coli*, *Enterococcus*, *S. aureus*, *coagulase-negative Staphylococci* on silver nanoparticle coated catheters was almost completely prevented [51]. However, silver nanoparticle could have genotoxic and cytotoxic effects on human cells at high dose [49]. Accelerated thrombin formation and platelet activation were also observed on surfaces of catheters coated with the silver nanoparticles, which could increase the thrombosis risk of patients in clinics [52]. As a result, much effort is still needed to improve the silver nanoparticle coating technology to diminish these side-effects.

Red alga *Delisea pulchra* produces halogenated furanones that can inhibit fouling of their surface. Furanones have been studied as a new class of anti-microbial agents [53,54]. Furanone was coated on biomaterial surfaces by physical adsorption and biofilm formation by *S. epidermidis* was significantly inhibited by furanone coating [55]. Furanone was also covalently bonded to Silastic Tenckhoff catheters and rendered inhibitory effect on biofilm formation [56]. Furthermore, in a sheep catheter infection model, furanone coated catheters tended to cause less severe infection than control catheters [56].

Covalently coupled 3-(trimethoxysilyl)-propyldimethyloctadecylammonium chloride (QAS) to silicone rubber will generate quaternary ammonium groups on the surface with antimicrobial activity. Viability of *S. aureus* adhered to QAS-coated silicone rubber was decreased, both *in vitro* and *in vivo* [57]. Quaternary ammonium functionalized silica nanoparticles was used to coat glass surfaces and exhibited inhibition of growth and accumulation of Gram-negative and Gram-positive bacteria on the surface [58].

One of the shortcomings of the bactericidal surfaces is that they could be covered by macromolecules and dead microorganisms, and then lose their antimicrobial function [59].

### 3.2. Anti-Adhesion Coating

The infection-resistant surface of indwelling medical devices could also be achieved by depositing a thin layer of anti-adhesion coating on the surface to reduce attachment of pathogenic bacteria. The number of bacteria that may adhere and their ability to grow and spread on biomaterial surfaces is greatly influenced by not only the bacteria but also the physicochemical properties of the biomaterial. The surface properties of biomaterials or medical devices can be changed by coating application or surface modification to create the desired anti-adhesion characteristics without altering the bulk properties of materials. These surface properties include chemical composition and reactivity, hydrophilicity and hydrophobicity [60], surface roughness [61,62] or texture [63], and surface charge. Following this approach, our research team has developed trimethylsilane (TMS) plasma nanocoatings using low temperature plasma coating technology to coat surfaces of stainless steel and titanium for reduced bacterial adhesion and biofilm formation [64]. Significant inhibition of *S. epidermidis* biofilm was observed on TMS plasma coated stainless steel and titanium. The biofilm inhibition could be attributed to the coating chemical inertness, low surface free energy, coating smoothness, and surface-bound CH_3_ groups. The changed surface properties could result in less protein adsorbed to the coated surfaces than that adsorbed to the uncoated stainless steel and titanium controls, leading to significantly decreased bacterial adhesion.

Harris *et al.* coated titanium surface with Poly(l-lysine)-grafted-poly(ethylene glycol) (PLL-g-PEG) to decrease non-specific adsorption of blood. The PEG coating also decreased *S. aureus* adhesion [65]. Zwitterionic poly(carboxybetaine methacrylate) (pCBMA) film grafted to glass surface was shown to be highly resistant to fibrinogen adhesion and *S. epidermidis* and *P. aeruginosa* attachment and accumulation [66]. It is believed that the surface hydration layer generated by these hydrophilic coatings could serve as a physical and energetic barrier to protein adsorption and thus bacteria adhesion [67].

A superhydrophobic coating on glass surface was synthesized from a mixture of nanostructured silica colloids and a low surface energy fluorinated silane xerogel. The adhesion of *S. aureus* and *P. aeruginosa* to the silica-colloid-doped fluorinated surfaces was decreased by two orders of magnitude *versus* the control [68]. It was found that fibrinogen adsorption on the superhydrophobic surface was very low, leading to low attachment of *S. aureus* [69]. Low surface energy chemistry and nano-textured morphology of the superhydrophobic coating could result in reduced protein adsorption and bacterial attachment. A barrier to wetting could be created by trapping pockets of air in the nano-scale morphology, which in effect presents a reduced surface area onto which protein molecules can diffuse from the solution [69].

The surface roughness of biomaterials has been recognized as one of many important factors for surface-bacterium interactions. Many studies have shown that the surface roughness of biomaterials strongly influences the degree of bacterial attachment to surfaces [62,70,71]. For instance, streptococcal adhesion was sensitive to surface roughness and enhanced as the roughness of composite surfaces increased from 20 nm to 150 and 350 nm [72]. *S. epidermidis* adhesion and growth were markedly higher on rough titanium surfaces than on smooth surfaces [73]. In contrast, there was greater attachment of *S. aureus* cells to mechanochemically polished titanium than the as-received titanium, even though the polished surfaces were much smoother. It was thus speculated that mechanochemical polishing generated nanoscale surface features on the titanium surfaces with a characteristic pattern more suitable for anchoring of spherical *S. aureus* cells [62].

Xu *et al.* reported that submicron (staphylococcal bacterial dimension) surface textures (400–500 nm) on poly(urethane urea) films reduced the material’s surface area accessible to bacteria of *S. epidermidis* and *S. aureus*, resulting in a decreased probability of interaction with the material surface or adhesive plasma proteins (e.g., fibrinogen and fibronectin) adsorbed onto the material. Thus, the flow of fluid over the material surface removes bacteria from a textured surface more efficiently than it would from a smooth surface, and subsequently resists bacterial adhesion and biofilm formation [63].

Organoselenium can catalyze the formation of superoxide radicals to prevent bacterial colonization on biomaterial surfaces [74]. Organoselenium antimicrobial agent selenocyanatodiacetic acid (SCAA) was coated on hemodialysis catheters by covalent bonding and demonstrated both *in vitro* and *in vivo* efficacy at preventing *S. aureus* biofilm formation [75].

Polymer brush coatings are another type of promising anti-adhesion coatings for inhibition of biofilms. Polymer brush coatings are formed when hydrophilic polymer long-chains are attached to a surface and stretch out into the surrounding medium [76,77]. Polymer brush coatings that have been mostly studied for preventing biofilm formation are made from poly(ethylene oxide) (PEO) [78–81]. As the PEO long-chains are highly mobile and attain extremely large exclusion volume, compression of the PEO long-chain brushes upon approach by incoming proteins or bacteria would give rise to an increase in the local concentration of PEO, which would lead to a repulsive osmotic pressure to repel the approaching proteins or bacteria and keep them away at a distance [77]. Excellent *in vitro* results have shown significant reduction in protein adsorption and bacterial adhesion, and thus the high effectiveness of polymer brush coatings in preventing bacterial adhesion [82,83]. In contrast, *in vivo* results [84,85] using PEO brush coatings have been discouraging mainly due to the weak surface attachment of polymer chains and the susceptibility of PEO to oxidation damage that prevent successful applications of such coatings for *in vivo* conditions [77].

Anti-adhesion coatings prevent biofilm formation at early stages, which should be more desirable in clinical settings. However, *in vivo* efficacy success is still elusive with many of the coatings. Due to the complexity of interaction between coating surfaces with bacteria and host proteins, the mechanism of anti-adhesion coatings is also difficult to pinpoint. As a result, more effort is needed to further exploit this promising strategy for prevention of biofilm related infections.

In summary, the bioengineering approaches (Table 2) could prevent biofilm formation which is more desirable than treating biofilm related infection. In spite of the shortcomings of many of the approaches, improving biomaterial anti-biofilm properties remains the most effective and promising strategy to prevent the morbidity and mortality associated with biofilm infections.

## 4. Conclusions

The approaches under development to prevent and treat biofilm caused infections include small molecules and matrix-targeting enzymes, bactericidal and anti-adhesion coatings. Small molecules and enzymes have been investigated to inhibit or disrupt biofilm formation. Anti-biofilm coatings have been targeting on modifying the surface of medical devices for enhanced inhibition of bacterial adhesion and/or growth leading to high resistance to biofilm formation. These novel anti-biofilm technologies could eventually lead to anti-biofilm therapies that are superior to the current antibiotic treatment.

## Figures and Tables

**Table 1:** Small molecules that can inhibit biofilm formation.

Agent	Mechanism	Effect	Reference
Anti-virulence compounds	Inhibition of gene expression of virulence factors	Inhibition of biofilm formation by *S. aureus*	[23]
Anti-biofilm compounds	Unknown	Inhibition of biofilm formation by *S. epidermidis*	[25]
ABC-1	Inhibition of c-di-GMP-inducible transcription	Inhibition of biofilm formation by multiple Gram-negative and Gram-positive bacterial pathogens	[26]
Aryl rhodanines	Unknown	Inhibition of biofilm formation by *S. aureus* and *S. epidermidis*	[29]
Cis-2-decenoic acid	Unknown	Dispersion of biofilms by *E. coli*, *K. pneumoniae*, *P. mirabilis*, *S. pyogenes*, *B. subtilis*, *S. aureus*, and *C. albicans*	[30]
d-amino acids	Unknown	Inhibition of biofilm formation by *S. aureus* and *P. aeruginosa*	[31]
*N*-acetylcysteine	Interference with exopolysaccharide formation in biofilms	Inhibition of biofilm formation by *S. epidermidis*	[32]
Chelators	Interference with metal ion’s function in biofilm formation	Inhibition of biofilm formation by *S. aureus*	[33]

**Table 2:** Surface modification approaches that can inhibit biofilm formation.

Coating agent	Coating method	Mechanism	Reference
Antibiotics	Non-covalent, covalent bonding	Bactericidal/Bacteriostatic	[40–44]
Silver	Plasma deposition, sol-gel coating, wet-chemical coating	Bactericidal	[46,47,51]
Furanones	Physical adsorption, covalent bonding	Bactericidal/Bacteriostatic	[55,56]
QAS	Covalent bonding	Inhibition of bacterial adhesion and viability	[57]
Silica nanoparticles with QAS	Covalent bonding	Bactericidal/Bacteriostatic	[58]
TMS	Plasma coating deposition with covalent bonding	Anti-adhesion	[64]
PLL-g-PEG	Physical adsorption & covalent coupling	Anti-adhesion	[65]
pCBMA	Zwitterionic surfaces grafted via radical polymerization	Anti-adhesion	[66]
Silica colloids/Silane xerogel	Synthesis of superhydrophobic coating	Anti-adhesion	[68]
Submicron surface textures	Physical surface roughness modification	Anti-adhesion	[63]
Selenocyanatodiacetic acid	Covalent bonding	Anti-adhesion	[75]
Polymer brush coatings	Surface grafting	Anti-adhesion	[82,83]
